# Molecular Cloning and Expression Analysis of Interleukin-8 and -10 in Yellow Catfish and in Response to Bacterial Pathogen Infection

**DOI:** 10.1155/2019/9617659

**Published:** 2019-06-16

**Authors:** Yingping Xiao, Lintian Yu, Guohong Gui, Yujie Gong, Xueting Wen, Wenrui Xia, Hua Yang, Long Zhang

**Affiliations:** ^1^State Key Lab Breeding Base for Zhejiang Sustainable Plant Pest Control, Institute of Quality and Standard for Agro-products, Zhejiang Academy of Agricultural Sciences, Hangzhou 310021, China; ^2^Guangxi Agricultural Vocational College, Nanning 530007, Guangxi, China; ^3^Institute of Ecology, Key Laboratory of Southwest China Wildlife Resources Conservation (Ministry of Education), China West Normal University, Nanchong 637009, China

## Abstract

The yellow catfish (*Pelteobagrus fulvidraco*) is an important economic freshwater aquaculture species in Asia. However, little is known about its immune response to bacterial pathogen infection. Here, two cytokines, the proinflammatory cytokine interleukin-8 (IL-8) and the anti-inflammatory cytokine interleukin-10 (IL-10), were identified and characterized in the yellow catfish for the first time. We found that the full length of the IL-8 cDNA was 784 bp and contained an open reading frame (ORF) of 336 bp, while the IL-10 gene was 973 bp in length with a 549 bp of ORF. In addition, both the IL-8 and the IL-10 had similar tissue-specific expression patterns. They were more abundant in the spleen and lowest expressed in the liver. Furthermore, IL-10 but not IL-8 was significantly upregulated in the intestine of yellow catfish by feed supplementation of* Clostridium butyricum* (CB). More importantly, the expression levels of intestinal IL-10 and IL-8 were up- and downregulated by pathogen* Aeromonas punctata* stimuli with the presence of CB, respectively. Collectively, these results suggest that IL-10 and IL-8 mediate important roles in the immunity of yellow catfish, and feed supplementation of CB may able to reduce the intestinal inflammation caused by bacteria infections through regulating the expression of IL-10 and IL-8.

## 1. Introduction

The yellow catfish (*Pelteobagrus fulvidraco*), one of the most important small freshwater aquaculture species, is well known for its excellent meat quality in Asian countries such as China, Japan, and South Korea [1]. Currently, the development of its farming is under the threatening of numerous diseases, which lead to a huge economic loss [2–7]. However, the molecular information of immune-related genes in yellow catfish is still poorly understood. Thus, to better understand the immune system as well as to enhance the pathogen resistance of the yellow catfish, it is essential to get more basal molecular information on this species.

Cytokines represent a group of small cell signaling proteins, peptides, or glycoproteins that function as effectors and regulators in immune response [8–11]. The signaling pathways provided by cytokines orchestrate the complex immune responses of many organisms. As a subset of cytokines, interleukins are also an important component in the intercellular regulation of the immune system [9–11]. So far, more than 40 interleukins, including inflammatory and anti-inflammatory mediators and lymphokines, have been found in vertebrates [12]. Interleukin-8 (IL-8), also known as CXCL8, is a member of the CXC chemokine family involved in inflammatory and immune response [13–16]. It has been reported as an effective adjuvant for vaccines in mammals and a potential immunopotentiator in vaccines against bacterial infections in fishes [17, 18]. Interleukin-10 (IL-10) is an important anti-inflammatory cytokine that has potent inhibitory effect on interleukin-2 and IFN-*γ* synthesis [19–22]. It has been known that IL-10 is able to enhance the activity of mast cells, B cells, and T cells and its expression can be regulated by multiple kinds of stimulation [8, 23, 24].

At present, the sequences of IL-8 and IL-10 have been reported in several fish species, including the mandarin fish, Atlantic cod, and channel catfish [25–29]. However, relatively few studies have investigated the sequence, expression pattern, and function of yellow catfish IL-8 and IL-10. In this study, we reported the full-length cDNA and conducted bioinformatics analysis of the two interleukin genes in the yellow catfish for the first time. Furthermore, we examined the expression pattern of these two genes in various tissues. Moreover, we investigated the effect of* Clostridium butyricum *(CB) on the regulation of IL-8 and IL-10 in* Aeromonas punctate *(AP) challenged yellow catfish. We concluded that the function of yellow catfish IL-8 and IL-10 genes may be similar to other species, and dietary CB supplementation is probably able to reduce the inflammation response in yellow catfish against AP infection through the regulation of IL-8 and IL-10.

## 2. Materials and Methods

### 2.1. Animals

Healthy juvenile yellow catfish were obtained from Nanchang Academy of Agricultural Science aquatic farm and maintained in 400 L circulation fiberglass tanks for 15-day acclimatization. After acclimatization, these 15-day-old yellow catfish were randomly divided into two groups of three replicates, each of 50 fish. As described previously [30], one group was provided with a standard commercial diet [31] and the other group was fed with 2×10^8^ CFU/g CB (Huijia Biotechnology Co. Ltd, Anji, China) in the basal diet. The temperature of the tank water was maintained at 26±2°C and pH at 5.5-6.0. Water was exchanged every 3 days at a rate of 30%. Fish were fed twice daily (8:00, 16:00) for 35 days at 2-4% of their body weight. After the 30-day feeding trial, two fish from each replicate (six fish per group) were randomly picked and the tissues, including the heart, liver, spleen, brain, gills, intestine, kidney, and muscle, were collected for RT-PCR. The remaining fish were then used for AP (Provided by Shanghai Ocean University) challenge experiment and continued with or without CB feeding. Briefly, the juveniles were exposed to AP by bath infection. The bacterial suspension was added to the tank water to give a final concentration of 10^6^ CFU/ml. Six fish were sampled at the time of challenge (control) and 24 h, 48 h, and 96 h after challenge, and the intestines of the fish were dissected. All of the samples were rapidly frozen in liquid nitrogen and stored at −80°C until total RNA extraction. All of the experiments were performed under the ethical guidelines of Zhejiang Academy of Agricultural Sciences for the care and use of laboratory animals (MS-222 was used for fish anesthetization).

### 2.2. RNA Extraction and cDNA Synthesis

Total RNA was isolated from various tissues of yellow catfish using TRIzol reagent (Invitrogen) and then transcribed into the first strand cDNA using the PrimeScript 1st Strand cDNA Synthesis Kit (Takara) according to the manufacture's instruction.

### 2.3. Reverse Transcription-Polymerase Chain Reaction (RT-PCR)

Primers were designed based on the conserved sequences of fishes. cDNA from the intestine of yellow catfish was used as template, and two cDNA fragments encoding the yellow catfish IL-8 and IL-10 were amplified by one round of PCR amplification, respectively. For IL-8, amplification was primed by primers DIL-8F and DIL-8R (Table 1), and the following program was used: 94°C for 4 min, 35 cycles of 94°C for 30 s, 48°C for 30 s, and 72°C for 10 min. For IL-10, the fragment was amplified by the degenerate primers DIL-10F and DIL-10R (Table 1), and the PCR program was the same as IL-8. The PCR products were subcloned into pGEM®-T plasmid (Promega) and sequenced, respectively. The obtained sequences were used to design gene-specific primers of the yellow catfish IL-8 and IL-10, respectively.

### 2.4. 5' and 3' Rapid Amplification of the cDNA Ends (5' and 3' RACE)

The first strand cDNAs for the 5' and 3' RACE were synthesized using First Choice® RLM-RACE Kit (Ambion), and used as templates to amplify the 5' and 3' region cDNA fragments of IL-8 and IL-10. KOD DNA Polymerase (Toyobo) was used to amplify 5' and 3' region of the IL-8 and IL-10. PCR was performed using the gene-specific primers (Table 1) and the RACE primers contained in the RACE Kit. For the 5' RACE of IL-8 and IL-10, the following program was used: 30 cycles of 5 s at 98°C and 30 s at 68°C. For the 3' RACE of IL-8, the following program was used: 30 cycles of 5 s at 98°C and 30 s at 66°C. The PCR program to amplify 3' region of IL-10 was 30 cycles of 5 s at 98°C and 30 s at 68°C.

### 2.5. Protein 3D Structure Prediction

The 3D structures of the proteins were predicted by I-TASSER server (http://zhanglab.ccmb.med.umich.edu/I-TASSER/) based on the homology structure modeling. Structural similarity of the two protein models was measured by TM-score and RMSD from I-TASSER server. The similarities between predicted structures and templates were superimposed by using PyMOL program (http://www.pymol.org/). The global and per-residue model quality were assessed using the C-scoring, which is a confidence score for estimating the quality of predicted models by I-TASSER. It was calculated based on the significance of threading template alignments and the convergence parameters of the structure assembly simulations. C-score is typically in the range of -5 to 2, where a C-score of higher value signifies a model with a high confidence and vice versa.

### 2.6. Phylogenetic Analysis

All of the amino acid sequences were aligned codon-to-codon as previously described [32, 33]. A neighbor-joining tree was constructed in Mega 7.0 [34] by calculating the* p*-distance among all sequences, and the reliability of each branch was tested by 1000 bootstrap replications.

### 2.7. Quantitative Analysis of the IL-8 and IL-10

For quantitative analysis of the gene expression, cDNA was synthesized from 2 *μ*g of total RNA from each sample using Prime Script 1st strand cDNA Synthesis Kit (Takara). An aliquot of 2 *μ*l was used as template in a 20 *μ*l RT-PCR reaction system. 18S rRNA was used as the reference gene. RT-PCR was performed in triplicate via SsoFast EvaGreen Supermix with CFX96 real-time PCR Detection System (Bio-Rad Laboratories). Data were analyzed with the CFX Manager™ software and were represented as means ± SEM. Expression levels of in different tissues were calculated relative to those in the liver using 18S as a reference gene, respectively. The primers used in the real-time quantitative for* IL-8 *are QIL-8F and QIL-8R, and for* IL-10 *are QIL-10F and QIL-10R (Table 1).

### 2.8. Statistical Analysis

Statistical analysis was performed using Student's t-test. *∗*, *∗∗*, and *∗∗∗* mean* p*<0.05,* p* <0.01, and* p* <0.001 compared to the controls, respectively.

## 3. Results

### 3.1. Molecular Cloning of IL-8 and IL-10 cDNAs from the Yellow Catfish

**Figure 1:**
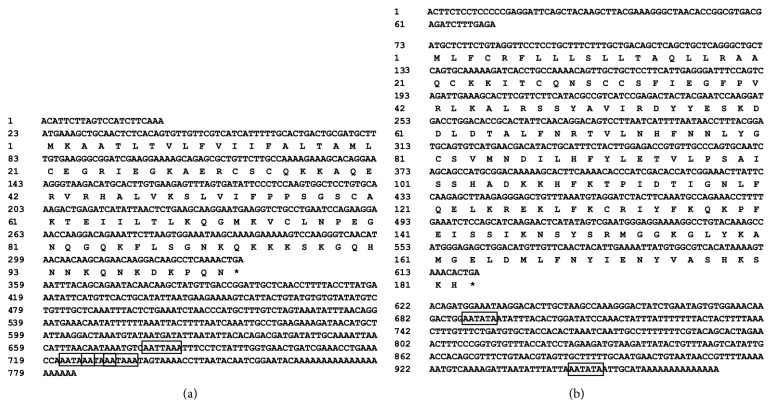
*Complementary cDNA and deduced amino acids sequences of IL-8 (a) and IL-10 (b) from the yellow catfish*. The polyadenylation signals are boxed and the asterisks (*∗*) represent the stop codons.

**Figure 2:**
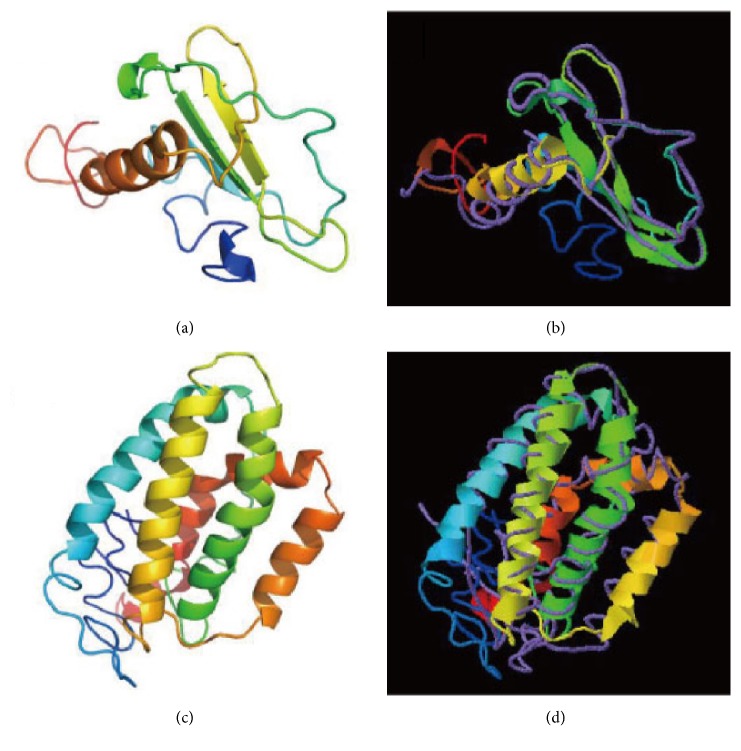
*The predicted 3D structure model and the main chain interface structure*. (a) Predicted 3D structure model of the yellow catfish IL-8. (b) Superimposed prediction model and native cartoon structures of the yellow catfish IL-8 and the human CXCL13. Rainbow structure represents the yellow catfish IL-8, while the purple line is the alpha carbon backbone of the human CXCL13. (c) Predicted 3D structure model of the yellow catfish IL-10. (d) Superimposed prediction model and native cartoon structures of the yellow catfish IL-10 and the human IL-20. Rainbow structure represents the yellow catfish IL-10, while the purple line is the alpha carbon backbone of the human IL-20.

The cDNA fragments of the IL-8 and IL-10 genes were obtained by RACE technology. The full-length cDNA sequences with deduced amino acids are shown in Figure 1. Briefly, the full length of IL-8 was 784 bp, containing a 336 bp of open reading frame (ORF); the 5' and 3' untranslated regions (UTR) were 22 bp and 426 bp, respectively. While the cDNA of IL-10 gene was 973 bp in length and contained a 72 bp of 5'UTR, 549 bp of ORF, and 352 bp of 3'UTR. The GenBank accession numbers of the IL-8 and IL-10 in the yellow catfish are KY218792 and KY218793, respectively. BLASTP search against the GenBank nonredundant database showed that IL-8 and IL-10 of the yellow catfish have the highest similarity (69% and 82%, respectively) to the corresponding orthologues of the channel catfish (*Ictalurus punctatus*), suggesting that these two genes were correctly identified. The predicted 3D structures of the IL-8 and IL-10 were highly similar to the human homologous (Figure 2), indicating that the functions of yellow catfish IL-8 and IL-10 genes may have similar immune functions to their counterparts in the human.

### 3.2. Phylogenetic Analysis of the IL-8 and IL-10 Genes

**Figure 3:**
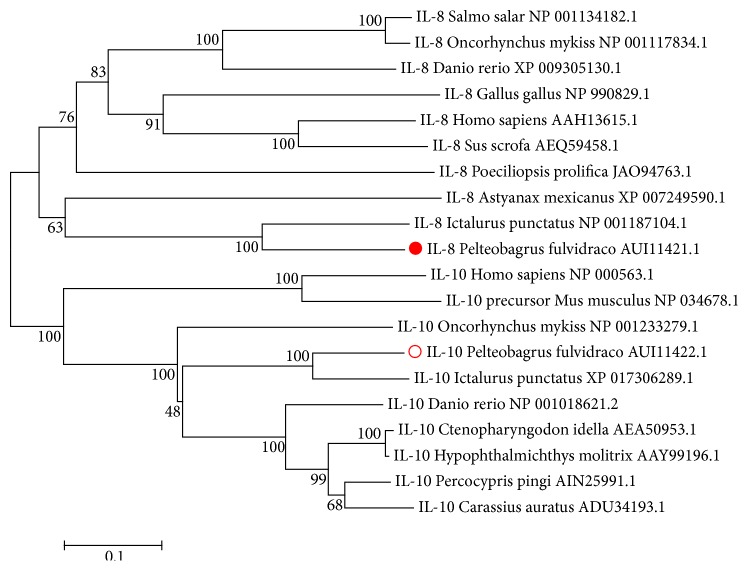
*The phylogenetic tree of the yellow catfish IL-8, IL-10, and the potentially related genes*. Protein sequences of the yellow catfish IL-8 and IL-10 were compared with IL-8 and IL-10 sequences from other representative species. The phylogenic tree was constructed by neighbor-joining method with 1000 bootstrap replicates. The scale bar is 0.1.

To assess the evolutionary relationships among vertebrate IL-8 and IL-10 genes, phylogenetic analysis was conducted using the neighbor-joining (NJ) method. The phylogenetic relationship demonstrates that the putative amino acid sequence of the yellow catfish IL-8 belongs to the conservative IL-8 family and shows a closely evolutionary relationship with the channel catfish (*Ictalurus punctatus*) IL-8 (Figure 3). Similarly, the deduced IL-10 protein sequence exhibited a closer relationship with IL-10s from different kinds of fishes (Figure 3).

### 3.3. Tissue Expression of the Yellow Catfish IL-8 and IL-10 Genes

**Figure 4:**
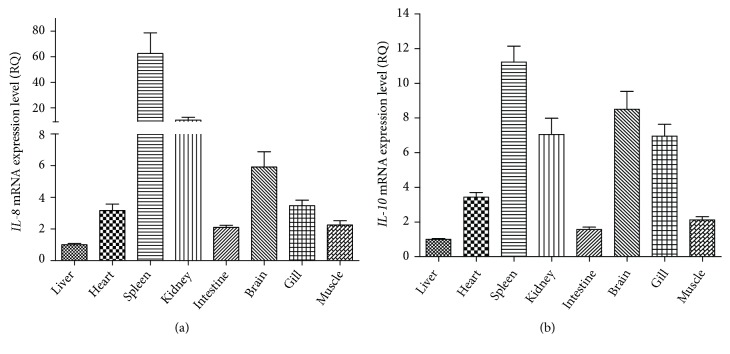
*Relative expression levels of IL-8 and IL-10 in different tissues of yellow catfish*. The relative mRNA level of IL-8 (a) and IL-10 (b) from different tissues of yellow catfish was quantified by RT-PCR. 18S gene served as the internal reference. Results were represented as mean ± SEM.

To evaluate the tissue expression patterns of IL-8 and IL-10 in yellow catfish, RT-PCR was performed in eight different tissues. As shown in Figure 4, the expressions of the IL-8 and IL-10 were detected in the liver, heart, spleen, kidney, intestine, brain, gill, and muscle. Furthermore, both genes were most abundantly expressed in the spleen, whereas the lowest expression level was detected in the liver.

### 3.4. Effect of CB Treatment on the Expression of IL-8 and IL-10 in Yellow Catfish

**Figure 5:**
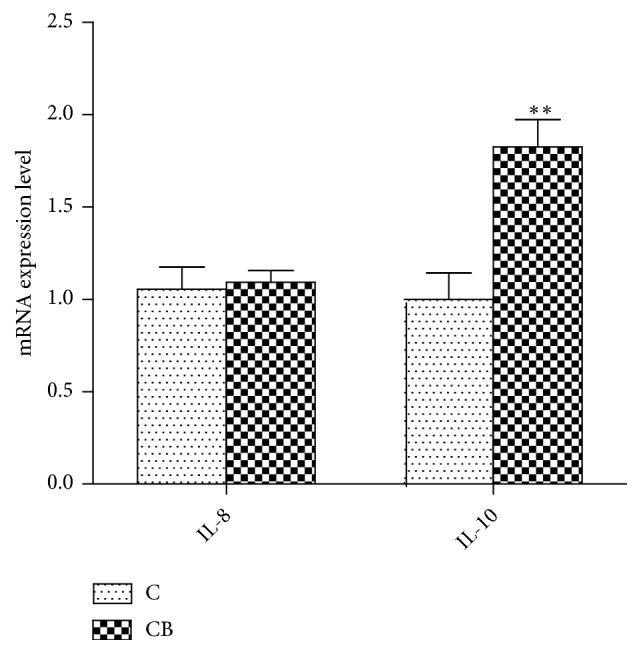
*Effect of Clostridium butyricum treatment on the expression of IL-8 (A) and IL-10 (B) in the intestine of yellow catfish*. C, control group; CB,* Clostridium butyricum* treated group. 18S gene served as the internal reference. Results were represented as mean ± SEM. *∗∗P* < 0.01.

We also studied the effect of dietary CB on the expression of the yellow catfish IL-8 and IL-10. As illustrated in Figure 5, the results showed that the expression of IL-8 in the intestine was barely changed after feeding with* CB*. Different from that of the IL-8, the intestinal IL-10 gene expression was significantly upregulated in the fish fed with CB (Figure 5(B)).

### 3.5. CB Supplementation Differently Regulates IL-8 and IL-10 Genes Expression in the Intestine of Infected Fish

**Figure 6:**
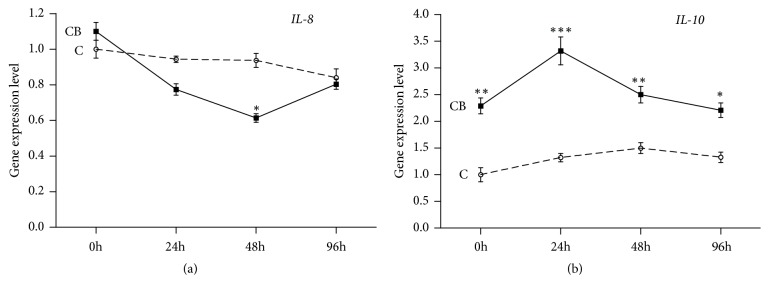
*Clostridium butyricum treatment differently regulated the expression of yellow catfish IL-8 (a) and IL-10 (b) challenged by Aeromonas punctata*. Fifteen-day-old yellow catfish were equally divided into two groups and fed with a standard commercial diet mixed with or without 2×10^8^ CFU/g* Clostridium butyricum *for 30 days. Fish were then exposed to* Aeromonas punctata *infection by bath infection for 24, 48, and 96 h, followed by RT-PCR analysis of the gene expressions of IL-8 and IL-10. C, control group; CB,* Clostridium butyricum* treated group. 18S gene served as the internal reference. Results were represented as mean ± SEM. *∗*:* P*<0.05; *∗∗*:* P*<0.01; *∗∗∗*:* P*<0.001.

To further evaluate whether CB is capable of regulating the expression of IL-8 and IL-10 in bacterial infection, we investigated the effect of feed supplementation of CB in yellow catfish challenged by AP. Yellow catfish fed with or without CB were sampled at the time of challenge (control), as well as at 24 h, 48 h, and 96 h after AP challenge. As shown in Figure 6, the stimulation of AP alone was not able to regulate the expression of IL-8 and IL-10 genes. However, with the presence of CB, the intestinal IL-8 mRNA abundance was significantly decreased at 48 h in the AP stimuli (Figure 6(a)). Conversely, the expression level of IL-10 in the intestine was significantly increased in the yellow catfish treated with AP and CB, peaking at 24h following the stimulation with AP in the presence of CB (Figure 6(b)).

## 4. Discussion

Bacterial infection is one of the most devastating problems in the fish farming. As the culture of yellow catfish in China has increased rapidly, understanding the innate immunity system of the yellow catfish is significant to enhance its resistance to pathogenic organisms and other environmental stresses. In this study, molecular cloning and gene expression analysis of IL-8 and IL-10 genes in yellow catfish were reported for the first time. The yellow catfish IL-8 and IL-10 genes exhibit similarity in gene structure and amino acid sequence to the counterpart sequences in other fishes, especially the channel catfish. Expression analysis of these two genes revealed a ubiquitous expression in all examined tissues, which suggested that both IL-8 and IL-10 serve critical roles in yellow catfish immunity. Furthermore, the expression patterns of IL-8 and IL-10 in yellow catfish were similar, with relatively high expression levels in the spleen, kidney, and gill as well as lower mRNA abundance in the muscle intestine and liver (Figure 4). Gene expression study in the Atlantic cod (*Gadus morhua*) also suggests the presence of IL-8 and IL-10 transcripts in the spleen, kidney, and gill [29]. However, for the puffer fish, IL-10 expression cannot be detected in the spleen and gill [35]. These results indicated that the tissue expression patterns of the interleukin gene family may differ in fishes, albeit they have close phylogenetic relationships.

Animals' production performance is affected by multiple factors [36, 37]. Recently, the beneficial effects of probiotic administration against bacterial pathogens and viral infections have been reported in aquaculture species [38–40]. CB, a butyric-acid producing and gram-positive bacterium, can elevate the tolerance of the intestine to pathogen invasion by inducing the secretion of anti-inflammatory cytokines such as IL-10 [41, 42]. An* in vitro* study showed that CB has preventive and therapeutic effects on* Salmonella enteritidis* and* Vibrio parahaemolyticus* infections in fish intestinal epithelial cells [43]. Furthermore, other studies indicated that CB can act as a potential probiotic to inhibit the growth of pathogens and prevent their colonization in fish intestinal tract [44]. For example, dietary supplementation of CB can mediate the immune response and improve the growth performance in the* Miichthys miiuy *[45]. Here, we investigated whether CB can regulate the expression of IL-8 and IL-10 in yellow catfish. Our results showed that feed supplementation of CB alone has no significant effect on the expression of IL-8 in the intestine of yellow catfish (Figure 5(A)). However, its expression in CB treated fish was decreased in response to AP infection (Figure 6(a)). Since probiotic bacteria have an anti-inflammatory effect in intestinal cells [38, 46], the downregulation of IL-8 gene expression is probably caused by a similar mechanism. The expression of intestinal IL-10 was also investigated in yellow catfish fed with or without CB. IL-10 expression in the intestine of yellow catfish is significantly increased by oral CB supplementation and further increased by the AP challenge (Figures 5(B) and 6(b)). These observations support previous reports that CB is able to achieve its beneficial effects by regulation of IL-10 expression [41, 42]. Together with the downregulation of IL-8 expression in CB treated fish, it is possible that CB plays an anti-inflammatory role in the intestine of yellow catfish. Since IL-10 can function by downregulating gene expression of other cytokines [47, 48], the opposite expression profiles (Figures 6(a) and 6(b)) may suggest a suppressive role for IL-10 on the transcription of IL-8. Taken together, oral supplementation of CB may be capable of alleviating the negative effects caused by AP infection.

In conclusion, we cloned and identified both the IL-8 and IL-10 genes from the yellow catfish in the present study. Furthermore, the expression patterns of these two genes in various tissues of yellow catfish were also investigated. Importantly, we examined the immune response of IL-8 and IL-10 genes to the probiotic bacteria CB and analyzed the effect of CB treatment on yellow catfish against AP infection. Our findings not only suggest that both IL-8 and IL-10 play critical roles in yellow catfish immunity but also provide a preliminary data for further development of effective disease control measures for the farming of yellow catfish.

## Figures and Tables

**Table 1:** Information of primers.

Primers	Sequences (5'-3')
DIL-8F	GGAAAAGCAGAGCGTTGTTT
DIL-8R	GACCTTCATTCCTTGCTTCA
DIL-10F	TTTAACTCVTTYGTKGAGRSHTTTCC
DIL-10R	TCVAGCTCYCCCATKGCTTT
3'RACEIL-8F	GTGGCTCCTGTGCAAAGACTGAGATCATA
5'RACEIL-8R	CAGGAGCCACTTGGAGGGAATATCACTA
3'RACEIL-10F	GCAATCAGCAGCCATGCGGACAA
5'RACEIL-10R	CGTAGTAGTCTCGGATGACGGCGTATGAA
QIL-8F	GAATGAAGGTCTGCCTGAATCCA
QIL-8R	CTGCTTGTTGTTATGTTGACCCTT
QIL-10F	GCATTTCTACTTGGAGACCGTGTT
QIL-10R	CGATGGTGTCGATGGGTGTTT
Q18sF	CGGACACGGAAAGGATTGACA
Q18sR	GGGCCGCGTAACTATTTAGCAT

## Data Availability

The data used to support the findings of this study are included within the article.
